# Attachment disturbance in women with depressive spectrum disorder, its connection with hostility

**DOI:** 10.1192/j.eurpsy.2024.1124

**Published:** 2024-08-27

**Authors:** S. N. Enikolopov, O. U. Vorontsova, T. I. Medvedeva, O. M. Boyko, I. V. Oleichik

**Affiliations:** ^1^Clinical psychology; ^2^Department of endogenous mental disorders and affective states, Federal Stare Budgetary Scientific Institution “Mental Health Research Center”, Moscow, Russian Federation

## Abstract

**Introduction:**

The results of the study of psychological factors of hostility in depression are presented. The topicality of the study is due to hostility considered, on the one hand, as a property of depression, and on the other hand, as a risk factor, associated with the likelihood of auto-aggressive behavior.

**Objectives:**

**The aim** of the study was to analyze the relationship between hostility and attachment disorders in endogenous depression.

**Methods:**

The study involved 49 patients with depressive disorder (mean age 19,8±4,5). All patients were assessed using the Hamilton Depression Rating Scale (HDRS-17 mean 21,03±6,02). All completed the following methods: Revised Experiences in Close Relationships (ECR-R); Symptom Check List-90-Revised (SCL-90R); Aggression Questionnaire by Buss and Perry (BPAQ); I-structural test by G. Ammon (ISTA). According to the “depression” parameter of the SCL-90R, the group was divided into subgroups with high and medium severity of depression. Analysis of variance (ANOVA) or Mann-Whitney test were. Correlation analysis (Spearman) and stepwise multiple regression analysis were also used.

**Results:**

At high levels of depression, the indicators of “hostility”, “destructive” and “deficit aggression” are statistically significantly higher. The severity of depression significantly correlates with the severity of “anxiety” in attachment (close relationships), as well as with pathological “narcissism”, “destructive external self-delimitation”, “deficient internal self-delimitation”.

For the measure of depression, regression analysis showed that the regression model explained more than 76% of the variance, with the measures of “interpersonal sensitivity”, “deficit narcissism”, and “avoidance” in attachment making significant contributions. For the “hostility” the regression model explains about 62% of the variance, while a significant contribution is made, as in the analysis of “depression”, by the indicators of “interpersonal sensitivity” and “avoidance”, however, unlike “depression”, the contribution of the “destructive narcissism” is noted in contrast to the “deficit narcissism”.

**Conclusions:**

With severe depressive symptoms, indicators of hostility are increased. Hostility in depression is associated with factors caused by a violation of early interpersonal relationships (anxious attachment), which causes increased sensitivity in relations with others, “building a barrier” between oneself and the external environment perceived as hostile in the narcissistic pathology, problems in emotional regulation. One of the targets of psychotherapeutic work may be the ambivalence between desire for symbiotic dependence and the experienced hostility.

**Disclosure of Interest:**

None Declared

